# Emotions Evoked by Colors and Health Functionality Information of Colored Rice: A Cross-Cultural Study

**DOI:** 10.3390/foods10020231

**Published:** 2021-01-23

**Authors:** Jin A Jang, Ji Eun Oh, Yeseul Na, Ga Eun Yeo, Mi Sook Cho

**Affiliations:** 1 Nutritional Science and Food Management Department, Ewha Womans University, Seoul 03760, Korea; geenna@hanmail.net (J.A.J.); chloe123@hanmail.net (Y.N.); sharpshock@naver.com (G.E.Y.); 2 College of Science & Industry Convergence, Ewha Womans University, Seoul 03760, Korea; oje96@ewha.ac.kr

**Keywords:** emotion measurement, emotion lexicon, emotion response, food, familiarity

## Abstract

This study aimed to examine the emotional responses evoked by cooked colored rice and its health functionality information in both consumers who eat rice as a staple food and consumers who do not eat rice as a staple food. Specifically, Korean and American consumers were exposed to colored rice and its health functionality information and an emotion lexicon was generated and measured based on focus group interviews (FGI) and two online consumer surveys. In test 1, the emotions evoked by presentation of stimuli to Koreans (*N* = 10) and Americans (*N* = 10) were extracted through FGIs and the first online consumer survey (Koreans = 69; Americans = 68) and an emotion lexicon was generated. As a result, a total of 34 terms were confirmed. Test 2 was conducted during the second online consumer survey (capturing data from a total of 208 Koreans and 208 Americans), utilizing the terms generated in test 1. In this test, only the colors (CO) of colored rice were presented to one group, while colors and health functionality information (CO&H) were presented to the other group. The overall liking for stimuli in both countries was highly correlated with familiarity. Koreans showed significantly more familiarity and liking for CO of white and black CO rice, while Americans showed significantly more familiarity and liking for CO of white and yellow rice. Hierarchical cluster analysis was performed to categorize the emotion terms, and the emotion terms were sorted into the three clusters, “Positive”, “Negative”, and “New”, for both countries. Under informed conditions, the emotions became more positive, and emotions in the “New” cluster were evoked in both countries. The current study employed a cross-cultural approach to assess consumers’ emotional responses to colored rice and health functionality information. Our findings suggest that providing foods with preferred colors for each culture and providing sufficient information on the said foods will help to promote unfamiliar foods.

## 1. Introduction

The color of food is one of the first attributes perceived and can evoke important emotions as well as flavor expectations [1,2], which can, in turn, influence consumer choices. The emotions evoked by food color may vary according to the cultural context in which the food is consumed [3]. Recent studies on the color of food have dealt mainly with multisensory perception, but very few studies have investigated the effect of the color of food from emotional and cultural viewpoints.

Intrinsic food factors such as nutritional information and ingredient labels may influence consumer expectations, which, in turn, influence sensory and emotional responses [4,5,6]. In particular, the provision of information on enhanced nutrition elicits positive emotions from consumers and contributes to positive product evaluation, thereby influencing consumers’ food selection decisions [7,8,9,10]. Spinelli et al. [5] reported that consumers’ emotional profiles are different under informed conditions, such as when information is provided via branding or packaging, compared to uninformed conditions. Therefore, it can be assumed that there are differences in the emotions evoked by the color of food based on whether or not health functionality information is provided. 

Although the term “emotion” might carry varying definitions, it is generally understood as an organism’s response to external stimuli, which can be relatively short and intense [11,12,13]. Emotion is different from other affective phenomena such as mood, preference, attitude, and even states of being [14,15]. Emotions related to food are similar to liking, such as pleasantness and satisfaction [16]. However, in the context of food-related emotions, studies have shown that emotional profiles provide additional information beyond liking and that food products can be distinguished more effectively using such information [16,17,18,19,20,21,22]. Emotions influence people’s eating behavior, including food choice, motivation to eat, and amount of food intake [10,23]. A growing body of consumers tend to choose foods that they can emotionally resonate with [24]; therefore, emotional connotation has become an important tool to differentiate food [10]. Accordingly, methodologies to measure the emotional responses of consumers in the context of products are being actively developed. Numerous methods, such as EsSense Profile^®^ [25], the Geneva Emotion and Odor Scale (GEOS) [26], EmoSemo [27], EmoSensory^®^ [28], and the Temporal Dominance of Emotions (TDE) [29], have been developed for general food evaluation. Moreover, emotion lexicons applied to specific food products are also being developed. Bhumiratana et al. [30] developed an emotion lexicon derived from the coffee experience, and Hu and Lee [31] developed Korean and Chinese coffee emotion lexicons. Ferrarini et al. [32] created an emotion lexicon specific to wine, and Chaya et al. [33] did the same for beer. Other product-specific emotion lexicons have also been developed, such as for dark chocolate [34], chocolate and hazelnut spreads [27], blackcurrant squash [21], and the color of hamburger packaging in a fast-food restaurant context [12].

According to the above, research measuring consumers’ emotional responses and product contexts is currently an active area, but research applying emotional measurement methods focusing on specific product contexts is still in its early stages, as the types of food studied to date are limited. Therefore, to utilize emotional response studies in the food industry, research into the various foods themselves and in the empirical context of foods that consumers encounter need to be carried out urgently. Additionally, no studies of staple foods such as cooked rice, which has strong food cultural characteristics, have been conducted. Furthermore, the questionnaires used to assess emotions were developed mostly in English-speaking countries and the emotion terms are therefore English in origin, but the same lexicon may not apply to different cultures and countries [35,36]. Therefore, culturally appropriate measurement tools should be used to allow subtle differences in the nuances of words to be detected [10]. 

Rice is one of the major staple crops worldwide, and it is a common staple throughout Asia, including in Korea [37,38,39]. With growing interest in gluten-free and ethnic foods, consumption of rice outside of Asia is increasing. In the U.S., rice consumption is steadily increasing; however, according to the FAO’s (Food and Agriculture Organization of the United Nations) Food Balance Sheet [40], as of 2011, rice consumption in Korea is 85.56 kg/capita/year, accounting for 51.1% of consumption of major cereal and root and tuber crops, while rice consumption in the U.S. is 7.47 kg/capita/year, accounting for only 4.6%. This indicates a wide gap between the two countries in terms of rice consumption. Recently, various colors of coated rice with enhanced functionality have been developed to promote consumption [41,42,43,44]. However, being newly developed, the products are not well-known amongst general consumers, and studies regarding how consumers perceive the various colors of rice are limited.

Therefore, this study aimed to (1) compare the emotions evoked by the colors of cooked colored rice between consumers who eat rice as a staple food and consumers who do not eat rice as a staple food and (2) compare how the presence vs. the lack of health functionality information for colored rice evoke different emotions. Accordingly, we identified and compared the emotions evoked by colors and health functionality information regarding cooked colored rice between American and Korean consumers in Korea. To do this, we first developed an emotion lexicon to measure the emotions evoked by exposure to colors of and health functionality information for cooked colored rice from consumers in each consumer group, and then, utilizing this lexicon, compared the emotions evoked in Koreans and Americans.

## 2. Materials and Methods

### 2.1. Experimental Design

We conducted two tests to generate a lexicon for stimulus-evoked emotions, and then, utilizing this lexicon, measured emotional responses. First, in test 1, the emotions evoked in Koreans and Americans by the colors and health functionality information of cooked colored rice were extracted via focus group interviews (FGI), and an emotion lexicon was confirmed through an online consumer survey (“first online consumer survey”) [30]. Subsequently, in test 2, we used the lexicon developed in test 1 and performed the second online consumer survey to investigate how the emotions evoked by cooked colored rice stimuli differed between Koreans and Americans.

### 2.2. Stimuli

In this study, the emotional responses to the colors (CO) and health functionality information (CO&H) of black, yellow, green, and red rice were evaluated. The colors were chosen based on the basic colors that could be distinctly distinguished from one another among the types of colored rice sold on the market. White rice was also included as a control. 

The color stimuli of the rice were presented as images, referencing the studies of Piqueras–Fiszman et al. [45,46,47] and Zhou et al. [48]. Before taking photos of the colored rice, the rice was prepared as follows. White and black rice was purchased from a grocery store located in Sinsu-dong Mapo-gu, Seoul, Korea, in May 2016, and colored rice coated with functional ingredients—*Monascus* (red), turmeric (yellow), and *Chlorella* (green)—were purchased in June 2016 via the online website of Nutry Rice Co. (https://www.hanarorice.com), a manufacturer of functional rice. Black rice samples were prepared by combining white and black rice and rinsing three times, and a 42:58 ratio of rice to water was used to cook the rice. The three colored rice samples were cooked in an electric pressure rice cooker (Cookoo, HJB0660SE, Seoul, Korea) at a ratio of 35:7:58 (white rice/colored rice/water). Nonblack colored rice was not rinsed because it was prewashed and coated with functional coloring materials. Immediately after cooking, the rice was gently stirred and mixed well with a rice scoop, 200 g of it was placed in a 250 mL round white ceramic bowl (10.5 × 5 × 6 cm), and photos were taken at the vertical and 45° angles using a CANON IXUS 160 camera. Cooked white rice (sticky japonica rice) was placed in a white ceramic bowl as per the cultures in which japonica rice is consumed, where cooked rice is usually served in a deep round bowl. 

The final images used in this study were all sized 891 × 444 pixels with a resolution of 96 dpi. The photos were arranged side by side for presentation. The CO and color information are provided in Supplementary Table S1. After sampling five colors from the colored rice using Photoshop 7.0 and obtaining the RGB values, we took the closest colors to these values from the Pantone^®^ color samples [49] available on the web and presented the color information in the form of a color palette based on the closest colors [50].

Health functionality information about the raw materials and coloring materials used for the colored rice samples was translated into Korean and English and provided along with images (CO&H) as stimuli. Images and the health functionality information of stimuli are shown in Table 1. Health functionality information was presented in the form of text box images with a pixel size of 891 × 228. Examples of the stimuli presented are shown in Supplementary Figure S1.

### 2.3. Test 1: Development of Emotion Lexicons

#### 2.3.1. Identifying Emotions

##### Participants

Researchers of the current study conducted FGIs to identify the various emotions and emotion terms evoked by the different colors of colored rice and its health functionality information. Interview participants were selected from among Koreans and Americans living in Seoul, Korea. The Koreans selected for this study ate rice as a staple more than three times a week, while the selected Americans did not eat rice as a staple food—they ate cooked rice less than once a month on average. To recruit participants, advertisements were posted on the bulletin boards of a language school in Ewha Womans University in Seoul and a U.S. military base located in Yongsan, Seoul, Korea, from 20 to 26 June 2016. Qualified applicants satisfying the requirements were selected. Based on the study conducted by Bhumiratana et al. [30] who utilized FGIs to develop an emotion lexicon for coffee beverages, we recruited a total of 20 people for this study: 10 Koreans and 10 Americans living in Korea. 

##### Data Collection Outline

Jiang et al. [10] proposed that to develop a lexicon for food, existing measuring tools for emotions should be used, and that complementary emotions regarding products should be obtained via consumer feedback collected using FGIs, after which these terms should be screened by means of statistical analysis. The current study generally followed the process developed by Jiang et al. [10], referencing the FGI process by Kim and Lee [51] and the lexicon development process by King and Meiselman [25] as well as by Bhumiratana et al. [30]. 

Our first step involved developing an emotion lexicon [19,20,30]. EsSense profile^®^ [25] is a tool for measuring emotions developed from practical usage, and it has been shown to have discriminative power across many food categories [19,20,50]. King et al. [19] contended that the list of emotion terms in the EsSense profile^®^ can be easily adapted and tailored to various products and product applications. We developed an emotion lexicon by modifying the EsSense profile^®^ taking into consideration the particular emotions felt about the colors and health functionality information of colored rice. The Korean version of EsSense profile^®^ was translated by three professional translators who are bilingual in English and Korean through the process of translation and back translation [35].

A total of six mini-focus groups were conducted during July and August 2016 in a soundproof meeting room at Ewha Womans University, Seoul, Korea. The participants were divided into six groups: three for Koreans and another three for Americans, with 3–4 participants per group [30]. The suggested group size for an FGI is six to eight; however, a smaller group size was chosen for the current study to provide sufficient time for each participant to respond to all nine stimuli. 

FGIs were conducted directly by the investigator in Korean for the Korean participants and in English for the Americans. The sessions took 60–85 min per group. Before starting an FGI, the study and the investigator were introduced to the participants. After participants had been fully informed of the purpose and content of the study, written signed consent was obtained. An FGI started with pre-established questions, while the questions and answers (Q&A) component was in the form of free talking [51]. The interview questions and procedures used for the FGIs are provided in Supplementary Table S2.

First, to determine the emotions of the participants concerning rice that they usually eat, they were asked what rice meant to them, their experience with rice and colored rice, and what they usually eat, i.e., consumption frequency, liking, and emotions. Images of the previously prepared white rice and four kinds of colored rice (red, yellow, green, and black) were then shown. The white rice image was shown first, followed by the four colored rice images in a random order in each group using a tablet PC (SM-T530, Samsung, Korea). After each image was shown, the participants were allowed to talk freely about the specific emotions they felt about each type of colored rice and what made them feel that way. Next, they were asked what emotions they felt about colored rice after they were provided with the CO&H and to talk about the differences between the emotions evoked by the CO&H and only by the CO. The questionnaire took the format of open-ended questions and complete interview sessions were recorded with prior participant consent. Nonverbal information that could not be recorded was summarized immediately after the interview and analyzed [51,52]. After the last question of the interview had been completed, a questionnaire containing the EsSense profile^®^ was presented using the “check all that apply” (CATA) format. The participants were asked to check the EsSense profile^®^ emotions that did not apply to any of the emotions they felt about the colored rice and health functionality information stimuli [30]. The interview was completed with a survey on general matters. After the interview was over, the participants were given a gift card worth 15,000 South Korean won (KRW) to compensate them for their time. 

##### Data Analysis

All the interviews were recorded, transcribed, and then analyzed [51]. Microsoft Word and Excel packages (Microsoft Corp., Redmond, WA, USA) were utilized for transcription and analysis, respectively. The emotion terms identified by participants during the interview were separately compiled and totaled together with the terms not excluded in the EsSense profile^®^ survey [30]. The emotion terms collected during the interview were reorganized in the process of analysis and translation. The main goals of the analysis and translation were to minimize the differences in the nuances of the emotion terms between the two languages, to determine the suitability of the colors and health functionality information, to increase the clarity of meaning, and to minimize redundancies [30,53]. The emotion terms were translated and back-translated into both Korean and English by three expert translators. To avoid redundancy, synonyms were grouped after consulting the Great Standard Korean Dictionary for Korean and the Microsoft synonym dictionary for English, and the term most suitable for clearly describing the emotion was selected. The remaining redundant terms were deleted. To shorten the survey time, low-frequency terms obtained by FGIs were deleted. 

#### 2.3.2. Selecting Terms 

##### The First Consumer Test

We conducted the first consumer survey to further develop the emotion lexicon to survey the emotions regarding the five types of colored rice [30]. We recruited both Korean and American participants for comparison. The Korean participants were recruited from Embrain, a local online research agency, and the American participants were recruited from Matrix Lap, an overseas partner of Embrain. A total of 137 participants (69 Koreans; 68 Americans) were recruited from 1 to 2 September 2016 via an online website. A total of 137 responses were included in the analysis.

The questionnaire used in this survey was modified and supplemented according to the purpose of this study. The survey took the form of CATA, in which stimuli were presented one by one to the participants and the participants were then asked to select all of the emotions evoked by stimuli from the given lexicon. The CATA terms were presented in English for the Americans and in Korean for the Korean participants [25,30]. First, an image of white rice was presented, and the group was asked to select via CATA questions all of the emotions evoked by the stimulus from among 60 emotion terms. The order of the terms was randomized. Subsequently, images of four different types of colored rice were presented one by one in random order, with each stimulus accompanied by CATA questions to be answered. Last, four kinds of CO&H stimuli corresponding to the four colored rice samples were presented in random order, and, similarly, each stimulus was accompanied by randomized CATA questions to be answered. 

### 2.4. Test 2: Emotional Response 

#### The Second Consumer Test

We used the same recruitment requirements as for the first survey to recruit participants for the second consumer survey. A total of 208 participants were recruited from each national population group. The participants were randomly assigned to two groups; a total of 107 Koreans and 105 Americans were assigned to group 1, and 101 Koreans and 103 Americans were assigned to group 2. A total of 416 valid questionnaires were used for analysis.

This second survey was carried out online from 27 to 28 September 2016. The questionnaires used in this survey were modified based on previous studies [20,25,30] and preliminary research. After a stimulus was presented, the overall liking, familiarity, and emotional responses were measured. First, white rice was shown to all groups as a control stimulus, then participants were asked to indicate their overall liking (using a 7-point hedonic scale: from 1 = extremely dislike to 7 = extremely like) [48] and familiarity (using a 5-point Likert scale: from 1 = not familiar at all to 5 = very familiar) and intensity for each of the 34 stimulus-evoked emotions (using a 5-point Likert scale: from 1 = not feeling it at all to 5 = feeling it extremely) [20,25,30]. The four types of colored rice were then shown with images to group 1, one by one in a random order, and four different CO&H stimuli were shown in the same way to group 2, followed by the same set of questionnaires mentioned above. 

**Figure 1:**
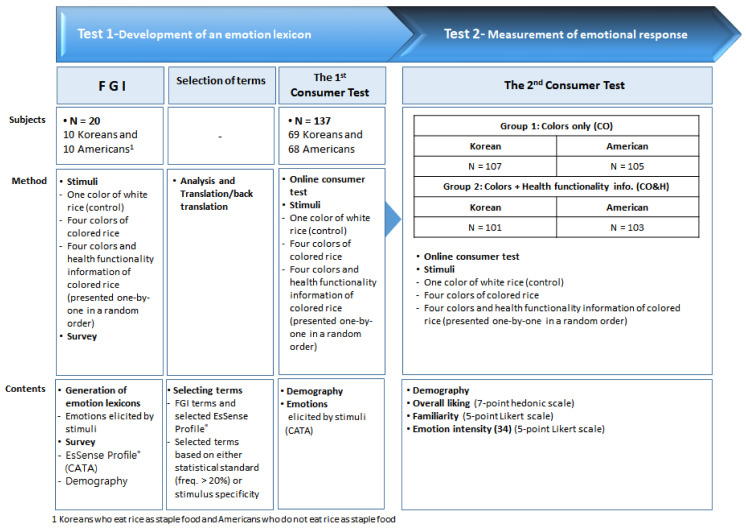
Process and methods of the study.

The survey procedures and methods are summarized in Figure 1. This study was conducted with the approval of the Institutional Review Board (IRB) at Ewha Womans University, regarding the purpose, content, and research methods for ethical consideration (IRB No. 118-4). 

### 2.5. Data Analysis

For the first study, we narrowed down the emotion terms using only the frequency of selection [25]. For the second experiment, to test for differences between the nine stimuli in overall liking and familiarity, a mixed-model ANOVA and Bonferroni multiple-comparison tests (*p* < 0.05) were performed. In addition, *t*-tests (*p* < 0.05) were carried out to detect differences in the health functionality information between groups. To assess the relationship between overall liking and familiarity and between overall liking/familiarity and emotion terms, Pearson’s correlation coefficients were calculated for mean overall liking scores and familiarity ratings, and for mean overall liking/familiarity scores and emotion lexicon ratings. To classify the 34 emotion terms elicited by the stimuli based on similarity, the emotion terms were classified into clusters using Ward’s method of hierarchical cluster analysis (HCA). To provide a further multivariate graphical representation of the emotion terms related to the stimuli, principal component analysis (PCA) was performed on the mean data of the significant emotion terms. SPSS 21.0 (SPSS Inc., Chicago, IL, USA) and XLSTAT (Addinsoft, Paris, France) were used for statistical analyses of the data.

## 3. Results and Discussion

### 3.1. Test 1: Development of Emotion Lexicons

#### 3.1.1. Identifying Emotions

Based on the FGIs, only the emotions evoked by stimulation with colors and health functionality information of the white and colored rice were screened and consolidated. The colored rice stimuli were presented as images by reference to studies of Piqueras–Fiszman et al. [45,46,47] and Zhou et al. [48].

Of the extracted terms, except for redundancies, a total of 46 terms (28 “positive” and 18 “negative”) were obtained for the Koreans, while 66 terms (34 “positive”, 22 “negative”, and 10 “unclear”) were obtained for the Americans. The emotion terms were grouped into three clusters, “negative”, “positive”, and “unclear”, and each emotion term was differentiated based on the reference studies conducted by Jiang et al. [10] and Laros and Steenkamp [54]. Among the 39 EsSense Profile^®^ emotion terms, two terms not evoked by the nine stimuli were deleted. The 46 terms obtained by the FGIs and the 37 EsSense Profile^®^ terms were extracted from the Korean participants. After removing 17 redundant terms, 66 remained. Likewise, 66 FGI terms and 37 EsSense Profile^®^ terms were extracted from the Americans. After removing 29 redundant terms, 74 remained. To decrease measurement errors in measuring the emotions evoked by the rice stimuli, cultural differences in the emotions surrounding rice needed to be taken into account, and linguistic differences needed to be minimized. Three experienced translators and interpreters fluent in both Korean and English performed translation and back translation of the 66 Korean terms and the 74 English terms. The emotion terms used by the Koreans and the Americans for rice stimuli were consolidated down to 60 terms through the translation process and are shown in Table 2. Of the 60 emotion terms, 32 were from the EsSense Profile^®^.

#### 3.1.2. Selecting Emotion Lexicons

To develop a tool to measure the emotions evoked in the American and Korean subjects by stimulation with CO and CO&H, the 60 emotion terms were analyzed. In most studies, high-frequency terms are usually selected; for instance, King and Meiselman [25] set the cut-off for checklist questions at 20%. Referencing King and Meiselman [25] and Bhumiratana et al. [30], terms that had both an average frequency of over 20% and distinguishable stimulus-specific emotions were selected. The final 34 emotion terms selected are presented in Table 3, of which 16 were EsSense Profile^®^ terms.

Kuesten et al. [55] pointed out that one of the main issues facing emotion studies during product development is an appropriate balance between positive and negative emotions. While conventional tools such as POMS (the Profile of Mood States) [56] and MAACL-R (the Manual for the Multiple Affect Adjective Check List) [57] tend to evaluate more negative emotions, newly designed measuring tools for commercial applications tend toward positive emotions [55]. For example, in the EsSense Profile^®^, 33 of the 39 terms are positive terms. Desmet and Schifferstein [23] also pointed out that there exists a “liking imbalance” or a positive bias in emotions toward many foods. However, consumer experience with products is not always positive, and negative emotions can provide meaningful information. The ratio of 18 “positive” to 11 “negative” terms in the Koreans and 19 to 11 in the Americans in this study was more balanced than that of the EsSense Profile^®^.

### 3.2. Test 2: Emotional Response

#### 3.2.1. Overall Liking and Familiarity 

Overall liking was examined in response to stimulation with the CO and CO&H of the colored rice, and the findings are shown in Table 4. First, among the Korean participants, there was a significant difference in the liking of the different colors of rice. When stimulated with the colored rice, Koreans liked the white and the black rice the most, while the lowest level of liking was found for red rice (*p* < 0.001). When the stimuli were presented with the CO&H, the white and the black rice were significantly liked, followed by yellow, green, and red rice in order (*p* < 0.001). When comparing presentation with CO and CO&H in the Korean participants, it was found that the only difference was the liking of yellow rice, which increased when health functionality information was provided (*p* < 0.01). Although there were no significant differences in the other stimuli, the average value of liking increased in the CO&H group relative to the CO group. 

Similarly to the Koreans, the Americans showed a distinct overall liking for specific rice colors. In the CO group, white rice was significantly preferred (*p* < 0.001), followed by yellow rice (*p* < 0.001), and no differences were found in the liking levels for the remaining three colored rice types. The results were similar in the CO&H group, but liking for black rice increased, leading to a difference in the overall ranking. White rice showed the highest level of liking, followed by yellow rice and black rice, but with no significant differences (*p* < 0.001). Additionally, there was no statistically significant difference observed between the two groups in the liking of stimuli; however, unlike the Koreans, the average liking scores for the colored rice were lower in the CO&H group than in the CO group, except for black rice. Although these results were not statistically significant, a reasonable assumption can be drawn that the health functionality information did not increase liking among the Americans.

Among the Koreans, health functionality information had no impact on the order of liking for the different rice colors. Additionally, among the Americans, no significant differences were observed between the two groups in the liking of stimuli. According to Spinelli et al. [5], under informed versus blind conditions, a difference in liking is influenced mainly by the perception of the sensory characteristics of products rather than the perception of the product brands. In particular, it was found that a difference in liking between the informed (CO&H) and blind (CO only) groups was observed only for the most preferred product under blind conditions; no other changes in liking were identified. These results are consistent with previous findings in the literature by Ng et al. [21]. Similar results were observed among the Koreans in this study who preferred yellow rice to a greater extent when presented with CO&H information instead of CO information. The reason for this is that *Curcuma* in yellow rice is an ingredient used in curry and is considered to be familiar to Koreans. In fact, in an FGI, one participant said he was reluctant to eat yellow rice because it looked like old rice when presented only as a color, but after finding out that it contains *Curcuma*, he said he would like to try it as it felt like curry. In Korea and Japan, curry is often eaten with rice, so it is believed that *Curcuma* is recognized as a good pairing to rice. In the case of black rice, which is commonly eaten in Korea, a high overall liking was shown under both informed and blind conditions, and no significant difference was observed between them. In the case of the Americans, yellow rice had the highest overall liking after white rice. Given that during the FGIs the American participants mentioned many specific foods such as “arroz con pollo” or “saffron” and “curry” when yellow rice was presented, we speculate based on these comments that yellow rice is familiar to Americans, and this is thought to have affected their liking.

The familiarity with colored rice based on CO and CO&H showed a similar pattern to that of liking in both the Koreans and the Americans (Table 5). The lower the familiarity with a color, the lower the level of liking, and vice versa. Pearson’s correlation coefficients for familiarity and overall liking for the stimuli were as high as 0.82 for the Koreans and 0.70 for the Americans (*p* < 0.001).

The Koreans presented with CO or CO&H information were most familiar with white rice, followed by black rice, and least familiar with red rice (*p* < 0.001 for both groups). Although not statistically significant, the CO&H group was slightly more familiar with black rice than the CO group, while their familiarity with yellow rice was significantly higher than that of the CO group (*p* < 0.05). The Americans in both the CO and CO&H groups were most familiar with cooked white rice, followed by yellow rice, and least familiar with red rice (*p* < 0.001 for both groups). The Koreans were familiar with black rice, while the Americans were unfamiliar with black rice, but more familiar with yellow rice. The Americans in the CO&H group reported significantly lower familiarity with green and red rice than the Americans in the CO group (*p* < 0.001).

In the present study, we assumed that the difference in overall liking for colored rice based on CO and CO&H might be influenced by familiarity with the stimuli in both countries. The results showed that familiarity scores based on CO and CO&H information were high in terms of ranking for black rice in the Koreans and yellow rice in the Americans, which is consistent with the order of overall liking for both groups. The Americans in the CO&H group reported significantly lower familiarity with green and red rice than the Americans in the CO group (*p* < 0.001). We speculate that the reason for this is that fortified types of rice coated with functional materials are unfamiliar to Americans. There was a significant difference in familiarity with green (*p* < 0.05) and red rice (*p* < 0.01) between the CO&H and CO groups (lower in the CO&H group), perhaps due to the participants’ unfamiliarity with the materials used to color rice. In support of this, during the FGIs in this study, some participants indicated that they were unfamiliar with the materials used to color rice (mainly *Monascus*). However, due to a significant increase in familiarity ranking for black rice by the Americans in the CO&H group (*p* < 0.001), and considering only the increase in the score of colored rice in overall liking, it is believed that familiarity with the functional ingredients of black rice affected the overall liking. Taken together, these results suggest that emotional responses were evoked by health functionality as well as by diverse factors such as individual preferences, familiarity with coloring materials, or ingredients.

Correlations between familiarity and emotion lexicons (Table 6) showed similar results as the correlations between overall liking and emotion lexicons, but when Pearson’s *r* value was classified using Cohen’s guideline [58] (small = *r* < 0.3; moderate = 0.3 < *r* < 0.5; strong = *r* > 0.5), the Americans generally showed lower *r* values than the Koreans and relatively lower *r* values than that for overall liking. This means that consumers of rice as a staple food have a stronger link between colored rice stimuli and emotions, while the opposite effect occurs for consumers who do not consume rice as their staple food. King and Meiselman [25] reported that emotional intensity increases as the frequency of product use increases, which is supported by the above results.

Meanwhile, among the correlations between familiarity and individual emotion lexicons, “bored” and “indifferent” represented a negative (–) correlation for the Koreans, but a positive (+) correlation for the Americans. It is assumed that this difference stems from the specificity of the Korean food culture, of which rice is characteristic. This means that Koreans show less “bored” or “indifferent” emotions regarding the familiarity of colored rice because they eat rice on a daily basis. The rice consumption of the Americans is not as frequent, so it is believed that they feel positive about colored rice, while “bored” and “indifferent” emotions also exist.

#### 3.2.2. Classification of the Emotion Lexicon

Using the 34 emotion terms generated earlier, the emotions evoked in the Korean and American participants by exposure to a total of nine CO & CO&H stimuli were measured and HCA was performed to categorize the emotion terms by country based on the similarity of the stimulus-evoked emotions [30,54] (Table 7). As a result, the emotion terms were sorted to the three clusters of “positive”, “negative”, and “new” for each country. In previous studies that used emotion terms, the terms were categorized as “positive”, “negative”, or “unclear” [10,21,30]; however, some of the CO&H information provided about the colored rice in this study was unfamiliar to the Koreans as well as the Americans—hence our categorization of the associated emotions as “new”. The “new” cluster comprised the following four terms: “adventurous”, “curious”, “different”, and “interested”; these words were commonly found among the participants from both Korea and America.

For the Koreans, 18 of the 34 emotion terms were clustered as “positive”, 11 as “negative”, and 5 as “new”. All of the emotion terms in the “positive” cluster showed a positive (+) correlation with liking, and those in the “negative” cluster showed a negative (–) correlation with liking (Table 8). All of the terms in the “new” cluster showed significantly positive (+) correlations with liking except for “different” (*p* < 0.001). For the Americans, the 34 emotion terms were classified as follows: 19 as “positive”, 11 as “negative”, and 4 as “new”. As a whole, the terms included in these clusters were similar to those for the Koreans; however, the term “special” was classified as “positive” for the Americans. 

All of the emotion terms in the “positive” cluster showed a significantly positive (+) correlation with liking (*p* < 0.001), while all of the terms in the “negative” cluster except for “bored” and “cautious” showed a significantly negative (–) correlation with liking. The terms “nostalgic” and “tame” which were categorized as “unclear” in previous studies [10,30] showed a significantly positive (+) correlation with liking and were included in the “positive” cluster, whereas “indifferent”, which showed a significantly positive (+) correlation with liking (*p* < 0.001), was included in the “negative” cluster. The other three terms in the “new” cluster showed significantly positive (+) correlations with liking (*p* < 0.001). In contrast, “different” showed no correlation with liking.

The emotion terms classified into the above three categories were generally similar between the Korean and American participants’ responses to colored rice stimuli. However, for the Koreans, “special” was a “new” emotion related to “curious” and “interested”, while for Americans, it was a “positive” emotion. In addition, “different” indicated a negative correlation in liking for the Koreans, but no correlation for the Americans. For the two countries, the emotion terms in the “new” cluster, which had a relatively low correlation between emotions and liking, are thought to be more meaningful than the other terms in that they provide new information on stimuli that are not explained by liking. Emotion terms such as “special”, “curious”, and “different” do not exist in the EsSense Profile^®^ and are specific emotion lexicon items applied to colored rice stimuli, which distinguish between Korean and American consumers.

In the aforementioned correlation between the familiarity and emotion lexicons, the Koreans—who consume rice on a daily basis—generally more strongly linked the colored rice stimuli with “positive”, “negative”, and “new” emotions based on familiarity. This tendency was similar to that of overall liking, showing similarity to the study by King and Meiselman [25]. As reported in the study of Ferdenzi et al. [59], consumers from different regions may also provide distinct emotional responses toward specific foods due to cultural significance. Based on the correlation between overall liking and the emotion lexicons, the emotions were more distinct for the Koreans than for the Americans, as in the case of “special” and “different”. This shows that the colored rice stimuli reflected not only the “frequency of use” of the consumers, but also the food cultural characteristics of rice, as Ferdenzi et al. pointed out [59].

#### 3.2.3. Emotion Profiles

PCA was performed to analyze the correlations between the CO&H of colored rice stimuli and the stimulus-evoked emotions in the Koreans and Americans, applying the average values of the 34 emotion terms to each of the nine stimuli [19,21,30]. 

**Figure 2:**
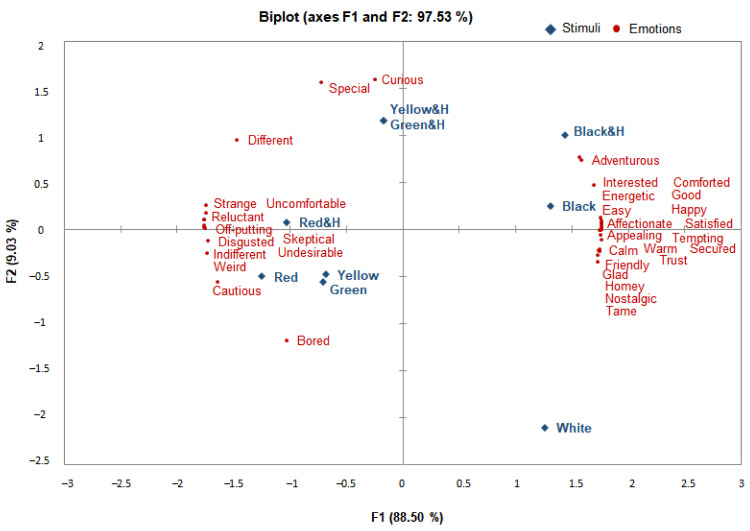
Principal component analysis (PCA) biplot showing the emotion profile elicited by the CO and CO&H of colored rice in the Korean respondents.

First, for the Koreans (Figure 2), the first two principal components (PCs) of the PCA accounted for 97.53% of the variance in the data (88.5% and 9.03%, respectively). As a whole, for PC1, the emotions in the “positive” cluster were concentrated in the positive (+) direction, while the emotions in the “negative” and “new” clusters tended toward the negative (–) direction. The emotions evoked by the CO and CO&H of black rice and the CO of white rice were positioned in the positive (+) direction of PC1, and the terms “adventurous”, “interested”, and “energetic” were concentrated predominantly around the CO and CO&H of black rice. Most of the “positive” terms, including “affectionate”, “appealing”, “calm”, “friendly”, “comforted”, “glad”, and “nostalgic”, were concentrated around the CO of black rice. The emotions evoked by the CO and CO&H of red, yellow, and green rice were positioned in the negative (–) direction of PC1. Ten terms, including “uncomfortable”, “strange”, “reluctant”, “off-putting”, “disgusted”, and “weird”, in the “negative” cluster were concentrated predominantly around the CO and CO&H of red rice, while emotions such as “curious” and “special” in the “new” cluster were concentrated around the CO&H of green and yellow rice. 

PC2 can be described as a dimension of high- and low-engagement/-activation emotions under the multidimensional circumplex model of emotion of Russell [60] and Watson and Tellegen [61]. Yellow and green CO&H were positioned in the positive (+) direction of PC2, and the terms “curious” and “special”, which are positive low-engagement/-activation emotions, were concentrated predominantly around them. The positive high-engagement/-activation emotions “energetic”, “affectionate”, and “satisfied” were positioned near the black CO. Toward the negative (–) direction of PC2, the yellow and green CO were positioned, and the negative low-engagement/-activation emotions “bored” and “cautious” were concentrated predominantly around them. The red CO and CO&H were positioned toward the positive position of PC2, and the negative high-engagement/-activation emotions “skeptical”, “reluctant”, and “weird” were positioned around them.

According to Spinelli et al. [62], the consumer cluster with the highest overall average liking score showed a higher frequency of use of emotion words associated with activation (energetic/excited and enthusiastic/inspired), whereas the cluster with the lowest average liking score showed a higher frequency of the use of emotion words associated with displeasure (unhappy/dissatisfied and blue/uninspired). Our findings mimic their findings: The CO of colored rice, except for black rice, was positioned in the negative (–) direction of PC2 associated with emotions in the “negative” cluster, while stimulation with CO&H resulted in a shift of emotions surrounding colored rice toward the positive (+) direction of PC2 and the “new” cluster. When participants were presented with the CO&H of black rice, they expressed emotions such as “interested” and “adventurous” in the “new” cluster. There was a big difference between the emotions evoked by CO only and by CO&H for yellow and green rice; while the emotions were mostly negative in response to CO stimulation, the emotions belonging to the “new” cluster became more dominant in response to CO&H stimulation. 

**Figure 3:**
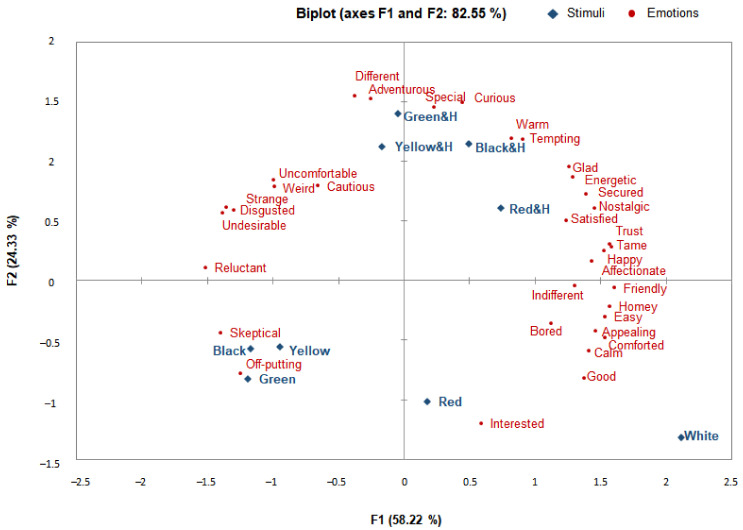
PCA biplot showing the emotion profile elicited by the CO and CO&H of colored rice in the American respondents.

For the Americans (Figure 3), the first two PCs of the PCA accounted for 82.55% of the variance in the data (58.22% and 24.33%, respectively). The emotions evoked by the CO&H of black rice, the CO and CO&H of yellow rice, and the CO of white rice were predominantly in the positive (+) direction of PC1. As a whole, most of the emotions in the “positive” cluster, e.g., “friendly”, “secured”, “nostalgic”, “trust”, “tame”, “happy”, and “affectionate”, were oriented in the positive (+) direction of PC1, while the emotions in the “new” cluster, such as “curious” and “interested”, were slightly positioned in the positive (+) direction of PC1. The emotions evoked by stimulation with the CO&H of red and green rice were slightly oriented toward the negative direction of PC1, while the emotions evoked by the CO of red, black, and green rice were concentrated in the negative (–) direction of PC1. Most of the emotions in the “negative” cluster, such as “reluctant”, “strange”, “disgusted”, and “undesirable”, were concentrated in the negative (–) direction. In contrast, the emotions in the “new” cluster, such as “different” and “adventurous”, were oriented slightly toward the positive direction.

As for the differences in the emotions depending on the provision of CO&H, under blind conditions (CO only), most emotions were positioned in the negative (–) direction and associated with negative emotions such as “skeptical” and “off-putting”, whereas under the informed conditions (CO&H), the emotions became more positive and emotions in the “new” cluster, such as “different”, “adventurous”, and “curious”, were evoked. Such emotional changes were the most prominent for cooked black, green, and red rice.

Under the multidimensional circumplex model of Russell [60] and Watson and Tellegen [61], the positive high-engagement/-activation emotions “tempting”, “energetic”, etc., were positioned near the black and yellow CO&H, while the positive low-engagement/-activation energy emotions “comforted”, “calm”, “easy”, “homey”, etc., were positioned near the white and yellow CO. Lastly, the negative high-engagement/-activation energy emotion “skeptical” and “off-putting” were positioned near the red, black, and green CO.

In the PCA, most of the positive emotions for the Korean group were found to be aroused by the CO and CO&H of black rice and the CO of white rice, while negative emotions were aroused by the CO and CO&H of red rice. For the Americans, the positive emotions were evoked by stimulation with the CO of white rice and the CO&H of yellow rice, while the negative emotions were evoked by the CO of black, red, and green rice. While previous research has mostly found that consumers tend to associate food products with positive emotion terms instead of negative terms [10,28,53], in the case of colored rice, the new food in this study, different results were observed. The stimuli with a low overall liking were associated with negative emotion terms and the stimuli with health functionality information were associated with “new” emotions. The results showed that the provision of CO&H to the Koreans resulted in the expression of more emotions in the “new” cluster, particularly for yellow and green rice. Similarly, the results of the American participants showed that low overall liking was associated with negative emotion terms, but CO&H evoked positive emotions in addition to emotions in the “new” cluster. These emotional changes were most prominent for black, green, and red rice. Emotional changes in response to the provision of health information were more marked among the Americans than among the Koreans. 

In summary, the emotions evoked by the CO and CO&H of colored rice among the Koreans and Americans were found to be different in terms of positive and negative emotions in PC1. For the Koreans, most of the positive emotions were concentrated dominantly in the CO and CO&H of black rice and the CO of white rice, in contrast to the negative emotions being concentrated dominantly in the CO and CO&H of red rice. For the Americans, the positive emotions were concentrated dominantly in the CO of white rice and the CO&H of yellow rice, in contrast to the dominant concentration of the negative emotions in the CO of black, red, and green rice. Given the differences in emotions provoked by the colored rice stimuli with the provision of CO&H, it can be reasonably deduced that the Koreans felt more “new” emotions under the informed conditions (CO&H). Among them, greater changes were observed for the yellow and green rice. For the Americans, the negative high-engagement/-activation emotions were linked to the CO of colored rice stimuli, but for the CO&H, the positive low-engagement/-activation emotions were linked with those in the “new” emotion cluster. These emotional changes were most evident for black, green, and red rice. The changes resulting from the provision of CO&H with colored rice were more prominent among the Americans in general than among the Koreans. Overall, for both the Koreans and the Americans, the principal components of the PCA had a strong explanatory power and finely distinguished between the stimuli and emotion lexicons. Therefore, the emotional measurement tools developed as a measure of the distinction between each stimulant and emotion using the developed emotion profile are considered valid.

## 4. General Discussion and Conclusion

In this study, using a measuring tool developed as part of a qualitative and quantitative cross-cultural study, the emotions evoked by the color and health and functionality information of colored rice were measured. For both Korean and the American consumers, the CO and CO&H stimuli evoked negative, positive, and new emotions, and most of them showed a significant correlation between overall liking and familiarity. In particular, the Koreans generally showed a higher correlation than the Americans in both categories, and more terms (mostly under the “new” cluster) were classified compared to those of the Americans. These results are thought to support the previous study of King and Meiselman [25] on the impact of frequency of product use.

The PCA showed that the negative, positive, and new emotions generated by the overall stimuli presented additional information about the stimuli that could not be otherwise explained by overall liking, which were more linked to emotions corresponding to the “new” cluster. For example, the Americans’ overall liking for colored rice stimuli was not significantly different when the CO&H was presented versus the CO alone; however, the emotion terms were more linked with “new” emotions when only the CO was presented compared to the CO&H condition. These results are consistent with the study of Ng et al. [21], in that emotional measurement can be a more specific measure of product differentiation than preferences.

This study showed that sensory factors such as taste are not the only determinants of food choice [63] and that emotions triggered by color can influence food liking. Emotions can ultimately affect food choices through physiological effects that change an individual’s appetite or behavior [64]. Although the application of emotion-based nutrition education has not been extensively researched, there is evidence showing that the emotion-based approach is able to effectively induce positive eating and physical activity [65]. Our findings suggest that emotional strategies for nutrition education should be applied differently by culture. Moreover, the use of emotion could be employed commercially. Our findings also suggest that providing foods with preferred colors for each culture and providing sufficient information on foods will help to promote unfamiliar food. 

The current study found that the familiarity with colored rice was highly correlated with overall liking, which was also highly correlated with emotional responses. Therefore, to promote colored rice, the promotion of colored rice needs to be carried out step by step with the preferred or familiar colored rice from each culture to increase familiarity with the said colored rice. For example, rice companies targeting American consumers could introduce yellow and black rice first, and then gradually introduce green and red rice. It is also thought that by emphasizing the health functional information in marketing, it will appeal to consumers’ “new” emotions such as “curious”, “interested”, and “special”. In particular, unlike the Americans in this study, “special” emotions evoked by colored rice were classified as “new” emotions for the Koreans, which could be used for marketing by evoking a “special” emotion in response to colored rice, although rice itself is a staple food. For Americans, providing information about the health functionality of colored rice will require a marketing strategy that provides more specific information about the product, as it is more linked to “new” emotions than “negative” emotions. Moreover, the use of familiar ingredients in each culture as health functional ingredients will likely be better accepted by consumers than the use of unfamiliar ingredients. 

We believe that our results are significant for the following reasons: First, consumer emotions evoked by food with cultural characteristics were examined, which has never been attempted before. In this study, using qualitative methodology in a cultural context, we generated an emotion lexicon in Korean and English to consider the peculiarities of the emotions evoked by rice among consumers who eat rice as a staple food and consumers who do not. Second, in this study, we extracted the emotions evoked by the appearance of cooked rice with a focus on color, as well as health functionality information. As a result, we were able to identify the consumer perceptions of colored rice that were not captured by liking, and we could identify the effect of health functionality information on consumer emotions toward colored rice. 

Nevertheless, this study has the following limitations: first, although this research studied Americans, who do not eat rice as a staple food, and the FAO’s Food Balance Sheet data [40] show that the Americans’ intake of rice is relatively lower than that of Koreans, it may be difficult to conclude that America is a non-rice-eating culture, as there are Americans who eat rice as a staple food. Second, our study included linguistic differences generated during the development of the emotion lexicon. When translating the emotion terms used in a heterogeneous group of people with different cultural and linguistic backgrounds, it is not easy to find vocabularies or expressions in two different languages that convey exactly the same meaning or nuance. Furthermore, some words or expressions that have distinct meanings in one language may not have such distinctions culturally or linguistically when translated into other languages. Therefore, differences in the meanings of the English and Korean emotion terms in our emotion lexicon used in this study may still exist. Third, a measurement tool was developed in this study through emotion lexicons derived from the colors of colored rice stimuli. CO&H and images including the color of the product are initial stimuli that have an important impact through packaging on purchasing a product. However, although the findings of this study can be applied to the purchasing stage of the product, applications in intake situations are limited. Therefore, future studies could explore the emotions presented upon consuming actual colored rice, which could then be compared with this study. 

When the Korean and American consumers in this study were presented with different colors and health functionality information of colored rice that they were unfamiliar with, emotions categorized as “new” dominated. Individual differences among consumers in terms of food neophobia and food acceptance can also influence stimulus-evoked emotions. Accordingly, it is recommended that further study should be undertaken to identify how emotions evoked by these consumer characteristics differ between different cultures. We addressed the emotional responses to the colors and health functionality information of colored rice using a cross-cultural approach, and our results can serve as a basis for emotional marketing to promote consumption and exportation of rice, including colored rice.

## Figures and Tables

**Table 1:** Images and ingredients and health functionality information of the stimuli.

Colors	Images	Ingredients and Health Functionality Information
White (control)	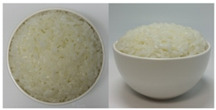	-
Yellow	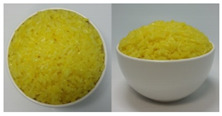	* Ingredients: • Short-grain white rice (Korea, pesticide-free), 33.47% • Powdered *Curcuma*, 0.013% • Water-soluble curcumin, 0.008% * *Curcuma aromatica* Slab. • *Curcuma* is originally from India and belongs to the ginger family. Since ancient times, *Curcuma* has been used as a spice, as a folk medicine for inflammation and skin problems, and as a coloring agent. • Curcuminoid, the main pigment in *Curcuma*, is known to inhibit cholesterol, arteriosclerosis, and inflammation. In addition, it has been reported that it is effective for various physiological activities and that it has anticancer, antioxidant, and anti-inflammatory effects.
Green	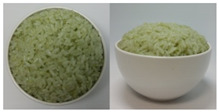	* Ingredients: • Short-grain white rice (Korea, pesticide-free), 33.46% • Powdered *Chlorella*, 0.024% * *Chlorella* • *Chlorella* is a kind of freshwater green algae and an excellent source of essential amino acids. It contains minerals including calcium, magnesium, iron, and zinc and vitamins such as inositol, niacin, ascorbic acid, carotene, and pyridoxine and is commonly used as a health food. • The *Chlorella* growth factor (CFG) contained in *Chlorella* is a biological active substance and is well-known for its bioactive functions, such as promoting growth in children, lowering cholesterol, and lowering blood pressure.
Red	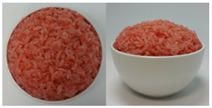	* Ingredients: • Short-grain white rice (Korea, pesticide-free), 33.47% • Powdered *Monascus* (Korean, total monacolin K, 0.4%), 0.01% * Red yeast • Red yeast rice is made by inoculating and cultivating *Monascus perpereus* in steamed rice. It has been used not only as a food, but also for coloring and taste enhancement of fermented foods such as alcohol for centuries in China. It is also famous for its effectiveness in promoting digestion and blood circulation. • The red pigment in red yeast rice has an anti-inflammatory effect and monacolin K has a cholesterol-lowering effect.
Black	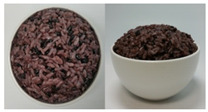	* Ingredients: • Short-grain white rice (Korea, pesticide-free), 28.27% • Short-grain black rice, 5.23% * Black rice • Black rice is a kind of colored rice. Since it is unpolished rice, it is high in dietary fibers, vitamins, and minerals. • Since black rice contains bioactive substances including polyphenols, flavonoids, anthocyanins, and γ-oryzanol, it is known for its antioxidant effects. • Anthocyanin, a pigment in the bran of black rice, is reported to have antibacterial, antimutagenic, thrombolytic, and anti-aging effects and also acts as an antioxidant.

**Table 2:** A list of the 60 emotion terms elicited by the nine stimuli in the focus group interviews (FGIs).

English	Korean	English	Korean	English	Korean
**Adventurous ^1,2^**	**도전해보고 싶은 ^+^**	Expectant ^+^	기대되는 ^+^	**Satisfied ^+^**	**만족스러운 ^+^**
**Affectionate ^+^**	**정성이 느껴지는 ^+^**	**Free ^+^**	**자유로운 ^+^**	Scared ^–^	무서운 ^–^
**Aggressive ^u^**	**자극적인 ^u^**	**Friendly ^+^**	**친근한 ^+^**	**Secure ^+^**	**안심할수있는 ^+^**
Appealing ^+^	호감이 가는 ^+^	Funny ^+^	재미있는 ^+^	Skeptical ^–^	의심스러운 ^–^
**Bored ^–^**	**지루한 ^–^**	**Glad ^+^**	**반가운 ^+^**	Special ^+^	특별한 ^+^
**Calm ^+^**	**진정되는 ^+^**	**Good ^+^**	**좋은 ^+^**	**Steady ^u^**	질리지않는 ^u^
Cautious ^–^	조심스러운 ^–^	**Guilty ^–^**	**죄책감이드는 ^–^**	Strange ^–^	이질감이드는 ^–^
Comfortable ^+^	편안한 ^+^	**Happy ^+^**	**행복한 ^+^**	Stressful ^–^	스트레스가생기는 ^–^
Comforted ^+^	위안이 되는 ^+^	Homey ^+^	집같은편안한 ^+^	**Tame ^u^**	**평범한 ^u^**
Confused ^–^	헷갈리는 ^–^	Indifferent ^u^	그저그런 ^u^	Tempting ^+^	구미가당기는 ^+^
Curious ^+^	호기심이 생기는 ^+^	Insecure ^–^	불안한 ^–^	**Tender ^+^**	부드러운 ^+^
**Daring ^u^**	**과감한 ^u^**	**Interested ^+^**	**관심이생기는 ^+^**	Trust ^+^	신뢰감이드는 ^+^
Different ^u^	색다른 ^u^	**Joyful ^+^**	**기쁜 ^+^**	Uncomfortable ^–^	부담스러운 ^–^
Disappointed ^–^	실망스러운 ^–^	**Loving ^+^**	**사랑스러운 ^+^**	**Understanding ^u^**	**이해되는 ^u^**
**Disgusted ^–^**	**혐오스러운 ^–^**	Melancholic ^–^	우울한 ^–^	Undesirable ^–^	원하지않는 ^–^
**Eager ^u^**	**간절히 원하는 ^u^**	**Mild ^u^**	순한 ^u^	**Warm ^+^**	**따뜻한 ^+^**
Easy ^+^	부담스럽지 않은 ^+^	**Nostalgic ^u^**	**향수가느껴지는 ^u^**	Weird ^–^	이상한 ^–^
**Energetic ^+^**	**에너지가 느껴지는 ^+^**	Off-putting ^–^	거부감이드는 ^–^	**Whole ^+^**	온전한 ^+^
**Enthusiastic ^+^**	**열정이 생기는 ^+^**	**Polite ^u^**	고상한 ^u^	**Wild ^u^**	날것의 ^u^
Excited ^+^	신나는 ^+^	Reluctant ^–^	주저하게되는 ^–^	**Worried ^–^**	걱정되는 ^–^

^1^ Bold terms are from the EsSense Profile^®^. ^2 +^ positive emotion, ^–^ negative emotion; ^u^ unclassified.

**Table 3:** Developed emotion lexicons.

English	Korean	English	Korean	English	Korean
**Adventurous ^1^**	**도전해보고 싶은**	**Friendly**	**친근한**	Skeptical	의심스러운
**Affectionate**	**정성이 느껴지는**	**Glad**	**반가운**	Special	특별한
Appealing	호감이 가는	**Good**	**좋은**	Strange	이질감이 드는
**Bored**	**지루한**	**Happy**	**행복한**	**Tame**	**평범한**
**Calm**	**진정되는**	Homey	집밥 같은 편안한	Tempting	구미가 당기는
Cautious	조심스러운	Indifferent	그저 그런	Trust	신뢰감이 드는
Comforted	위안이 되는	**Interested**	**관심이 생기는**	Uncomfortable	부담스러운
Curious	호기심이 생기는	**Nostalgic**	**향수가 느껴지는**	Undesirable	원하지 않는
Different	색다른	Off-putting	거부감이 드는	**Warm**	**따뜻한**
**Disgusted**	**혐오스러운**	Reluctant	주저하게 되는	Weird	이상한
Easy	부담스럽지 않은	**Satisfied**	**만족스러운**		
**Energetic**	**에너지가 느껴지는**	**Secure**	**안심할 수 있는**		

^1^ Bold terms are from the EsSense Profile^®^.

**Table 4:** Overall liking according to the colors and health functionality information of colored rice in Korean and American respondents.

		White	Yellow	Green	Red	Black	F value
**Koreans**	CO	4.95 ± 1.14 ^d^	3.28 ± 1.30 ^b^	3.28 ± 1.47 ^b^	2.47 ± 1.29 ^a^	5.32 ± 1.07 ^d^	99.23 ***
	CO&H	5.20 ± 1.06 ^d^	3.91 ± 1.37 ^c^	3.51 ± 1.29 ^b^	2.82 ± 1.33 ^a^	5.43 ± 1.18 ^d^	80.38 ***
	*t* value	−1.6	−3.4 **	−1.17	−1.9	−0.76	-
**Americans**	CO	5.09 ± 1.28 ^c^	4.26 ± 1.54 ^b^	3.61 ± 1.72 ^a^	3.74 ± 1.73 ^a^	3.60 ± 1.69 ^a^	16.45 ***
	CO&H	5.02 ± 1.20 ^c^	4.09 ± 1.62 ^b^	3.38 ± 1.64 ^a^	3.44 ± 1.60 ^a^	3.82 ± 1.71 ^a,b^	18.61 ***
	*t* value	0.39	0.77	0.99	1.33	−0.92	-

Notes: CO: five different colors of rice; CO&H: color and health functionality information on four types of colored rice and white rice (control). Values within rows with different lowercase letters are significantly different according to the Bonferroni multiple comparisons test with a significance level of *p* < 0.05. ** Significant differences between the means of the overall liking between CO and CO&H at *p* < 0.01; *** significant differences between the means of the emotion terms elicited by the five stimuli using the mixed model ANOVA procedure at *p* < 0.001.

**Table 5:** Familiarity according to the colors and health functionality information of colored rice in the Korean and American groups.

		White	Yellow	Green	Red	Black	F Value
**Koreans**	CO	3.87 ± 0.95 ^c^	1.88 ± 1.06 ^b^	1.94 ± 1.08 ^b^	1.34 ± 0.93 ^a^	3.9 ± 50.79 ^c^	219.77 ***
	CO&H	4.01 ± 0.79 ^d^	2.29 ± 1.25 ^c^	1.94 ± 0.98 ^b^	1.34 ± 0.84 ^a^	4.08 ± 0.76 ^d^	206.35 ***
	*t* value	−1.2	−2.55	−0.01	0.42	−1.24	-
**Americans**	CO	3.56 ± 1.04 ^c^	2.61 ± 1.27 ^b^	2.18 ± 1.27 ^a^	2.20 ± 1.35 ^a^	2.27 ± 1.31 ^a,b^	39.79 ***
	CO&H	3.61 ± 0.99 ^d^	2.30 ± 1.29 ^c^	1.84 ± 1.12 ^a,b^	1.63 ± 1.21 ^a^	2.07 ± 1.27 ^b,c^	65.93 ***
	*t* value	−0.39	1.79	2.01 *	3.19 **	1.15	-

Notes: CO: five different colors of rice; CO&H: color and health functionality information on four types of colored rice and white rice (control). Values within rows with different lowercase letters are significantly different according to the Bonferroni multiple comparisons test with a significance level of *p* < 0.05. Significant differences between the means of the overall liking between CO and CO&H at * *p* < 0.05 and ** *p* < 0.01; *** significant differences between the means of the emotion terms elicited by the five stimuli using the mixed model ANOVA procedure at *p* < 0.001.

**Table 6:** Correlations between the familiarity and emotion lexicon items elicited by the stimuli in the Korean and American groups.

Emotion	Koreans	Emotion	Americans
Adventurous ^N^	0.471 ***	Adventurous ^N^	0.082 **
Curious ^N^	0.106 **	Curious ^N^	0.123 ***
Different ^N^	–0.366 ***	Different ^N^	–0.140 ***
Interested ^N^	0.426 ***	Interested ^N^	0.344 ***
Special ^N^	–0.061 *	Special ^+^	0.288 ***
Affectionate ^+^	0.684 ***	Affectionate ^+^	0.409 ***
Appealing ^+^	0.735 ***	Appealing ^+^	0.568 ***
Calm ^+^	0.687 ***	Calm ^+^	0.450 ***
Comforted ^+^	0.694 ***	Comforted ^+^	0.562 ***
Easy ^+^	0.545 ***	Easy ^+^	0.477 ***
Energetic ^+^	0.531 ***	Energetic ^+^	0.342 ***
Friendly ^+^	0.844 ***	Friendly ^+^	0.478 ***
Glad ^+^	0.783 ***	Glad ^+^	0.448 ***
Good ^+^	0.749 ***	Good ^+^	0.519 ***
Happy ^+^	0.726 ***	Happy ^+^	0.461 ***
Homey ^+^	0.807 ***	Homey ^+^	0.491 ***
Nostalgic ^+^	0.709 ***	Nostalgic ^+^	0.467 ***
Satisfied ^+^	0.732 ***	Satisfied ^+^	0.530 ***
Secured ^+^	0.712 ***	Secured ^+^	0.444 ***
Tame ^+^	0.672 ***	Tame ^+^	0.562 ***
Tempting ^+^	0.683 ***	Tempting ^+^	0.451 ***
Trust ^+^	0.672 ***	Trust ^+^	0.471 ***
Warm ^+^	0.661 ***	Warm ^+^	0.458 ***
Bored ^–^	–0.104 ***	Bored ^–^	0.137 ***
Cautious ^–^	–0.211 ***	Cautious ^–^	0.022
Disgusted ^–^	–0.493 ***	Disgusted ^–^	–0.136 ***
Indifferent ^–^	–0.304 ***	Indifferent ^–^	0.227 ***
Off-putting ^–^	–0.604 ***	Off-putting ^–^	–0.102 **
Reluctant ^–^	–0.654 ***	Reluctant ^–^	–0.170 ***
Skeptical ^–^	–0.609 ***	Skeptical ^–^	–0.205 ***
Strange ^–^	–0.653 ***	Strange ^–^	–0.247 ***
Uncomfortable ^–^	–0.592 ***	Uncomfortable ^–^	–0.178 ***
Undesirable ^–^	–0.604 ***	Undesirable ^–^	–0.180 ***
Weird ^–^	−0.681 ***	Weird ^–^	−0.271 ***

Notes: Emotion terms elicited by the nine stimuli are clustered as “new” (N), “positive” (+), and “negative” (–). Pearson’ s correlation coefficient (*r*) with significance level of * *p* < 0.05, ** *p* < 0.01, and *** *p* < 0.001.

**Table 7:** Classification of the emotion lexicons in the Korean and American groups.

Koreans			Americans		
Cluster 1	Cluster 2	Cluster 3	Cluster 1	Cluster 2	Cluster 3
“New”	“Positive”	“Negative”	“New”	“Positive”	“Negative”
Adventurous	Affectionate	Bored	Adventurous	Affectionate	Bored
Curious	Appealing	Cautious	Curious	Appealing	Cautious
Different	Calm	Disgusted	Different	Calm	Disgusted
Interested	Comforted	Indifferent	Interested	Comforted	Indifferent
Special	Easy	Off-putting		Easy	Off-putting
	Energetic	Reluctant		Energetic	Reluctant
	Friendly	Skeptical		Friendly	Skeptical
	Glad	Strange		Glad	Strange
	Good	Uncomfortable		Good	Uncomfortable
	Happy	Undesirable		Happy	Undesirable
	Homey	Weird		Homey	Weird
	Nostalgic			Nostalgic	
	Satisfied			Satisfied	
	Secured			Secured	
	Tame			Special	
	Tempting			Tame	
	Trust			Tempting	
	Warm			Trust	
				Warm	
5	18	11	4	19	11

**Table 8:** Correlations between overall liking and the emotion terms elicited by the stimuli in the Koreans and the Americans.

Emotions	Koreans	Emotions	Americans
Adventurous ^N^	0.61 ***	Adventurous ^N^	0.28 ***
Curious ^N^	0.28 ***	Curious ^N^	0.35 ***
Different ^N^	−0.21 ***	Different ^N^	0
Interested ^N^	0.55 ***	Interested ^N^	0.57 ***
Special ^N^	0.10 ***	Special ^+^	0.43 ***
Affectionate ^+^	0.77 ***	Affectionate ^+^	0.50 ***
Appealing ^+^	0.80 ***	Appealing ^+^	0.74 ***
Calm ^+^	0.71 ***	Calm ^+^	0.51 ***
Comforted ^+^	0.72 ***	Comforted ^+^	0.64 ***
Easy ^+^	0.57 ***	Easy ^+^	0.53 ***
Energetic ^+^	0.63 ***	Energetic ^+^	0.48 ***
Friendly ^+^	0.80 ***	Friendly ^+^	0.60 ***
Glad ^+^	0.78 ***	Glad ^+^	0.58 ***
Good ^+^	0.78 ***	Good ^+^	0.68 ***
Happy ^+^	0.78 ***	Happy ^+^	0.61 ***
Homey ^+^	0.79 ***	Homey ^+^	0.59 ***
Nostalgic ^+^	0.68 ***	Nostalgic ^+^	0.45 ***
Satisfied ^+^	0.78 ***	Satisfied ^+^	0.68 ***
Secured ^+^	0.75 ***	Secured ^+^	0.55 ***
Tame ^+^	0.61 ***	Tame ^+^	0.61 ***
Tempting ^+^	0.76 ***	Tempting ^+^	0.64 ***
Trust ^+^	0.73 ***	Trust ^+^	0.61 ***
Warm ^+^	0.70 ***	Warm ^+^	0.57 ***
Bored ^−^	–0.19 ***	Bored ^–^	–0.02
Cautious ^−^	–0.21 ***	Cautious ^–^	–0.04
Disgusted ^−^	–0.55 ***	Disgusted ^–^	–0.32 ***
Indifferent ^−^	–0.35 ***	Indifferent ^–^	0.15 ***
Off-putting ^−^	–0.64 ***	Off-putting ^–^	–0.24 ***
Reluctant ^−^	–0.67 ***	Reluctant ^–^	–0.28 ***
Skeptical ^−^	–0.62 ***	Skeptical ^–^	–0.27 ***
Strange ^−^	–0.63 ***	Strange ^–^	–0.28 ***
Uncomfortable ^−^	–0.61 ***	Uncomfortable ^–^	–0.31 ***
Undesirable ^−^	–0.66 ***	Undesirable ^–^	–0.35 ***
Weird ^−^	−0.67 ***	Weird ^–^	−0.33 ***

Notes: Emotion terms elicited by the nine stimuli and clustered as “new” (N), “positive” (+), and “negative” (–). Pearson’ s correlation coefficient (*r*) with significance level of * *p* < 0.05, ** *p* < 0.01, and *** *p* < 0.001.

## Data Availability

The data presented in this study are available on request from the corresponding author. The data are not publicly available due to the institutional data policy.

## Supplementary Materials

The following are available online at https://www.mdpi.com/2304-8158/10/2/231/s1, Figure S1: Examples of stimuli: (a) color (CO); (b) color and health functionality information (CO&H), Table S1: Reference of colors in the stimuli, Table S2: Focus group interview (FGI) questions and procedures.
