# Supplementary material for: Emotions Evoked by Colors and Health Functionality Information of Colored Rice: A Cross-Cultural Study
Source: Foods. 2021 Jan 23;10(2):231. doi: 10.3390/foods10020231 (PMC7912385; doi:10.3390/foods10020231)
Supplement: Supplementary file 1 [file foods-10-00231-s001.pdf]

Supplementary Table S1. Reference colors of the stimuli.

Stimuli	Color pallet
	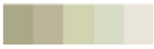
	PANTONE® 452 U
White	PANTONE® 2323 C
(control)	PANTONE® 5803 UP
	PANTONE® 9585 C
	PANTONE® P 169-1U
	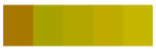
	PANTONE® 126 C
	PANTONE® 391 XGC
Yellow	PANTONE® 398C
	PANTONE® P 2-16.0
	PANTONE® 7765 XGC
	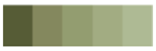
	PANTONE® 7763 C
	PANTONE® 7749 UP
Green	PANTONE® 5773 CP
	PANTONE® 5783 C
	PANTONE® 5793 XGC
	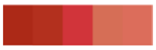
	PANTONE® 2443 CP
	PANTONE® 484 C
Red	PANTONE® 1805 CP
	PANTONE® 10150 C
	PANTONE® 7598 U
	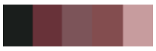
	PANTONE® 419 C
Black	PANTONE® 504 CP
	PANTONE® P78-1311

PANTONE® 8565 C

PANTONE® P 67-2C

Supplementary Table S2. Focus group interview (FGI) questions and procedures.

Run time	Total time	Interview questions and procedures
10 min	10 min	Introduction:<br>• Greetings and investigator introduction<br>• Research introduction and consent
15 min	25 min	Meaning of rice:<br>Experiences and emotions about rice:<br>• How often does the interviewee consume rice?<br>• Does the interviewee like or dislike rice?<br>• What does the interviewee feel when seeing their most and least favorite rice?<br>Experiences and emotions about colored rice:<br>• Has the interviewee ever experienced colored rice? How often?<br>• What colors of rice have the interviewee experienced and how did they feel abou<br>t it?
20 min	45 min	Emotions elicited by stimuli:<br>• Are any emotions elicited by the stimuli and why?<br>- Presentation of an image (white)<br>- Presentation of four images (four stimuli in a random order)<br>- Presentation of four images and health functionality information<br>(four stimuli except white in a random order)
10 min	55 min	EsSense Profile®:<br>• Emotions listed in the EsSense Profile® not elicited by any of the stimuli
5 min	60 min	Simple survey and interview closing statements

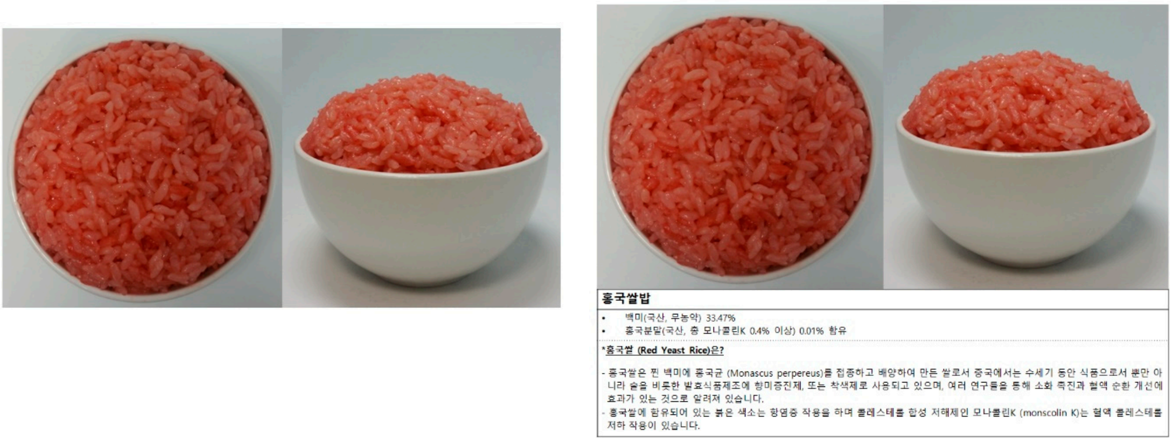

(a) (b)

Supplementary Figure S1. Examples of stimuli: (**a**) color (CO); (**b**) color and health functionality information (CO&H).
